# Exploring aryl hydrocarbon receptor expression and distribution in the tumor microenvironment, with a focus on immune cells, in various solid cancer types

**DOI:** 10.3389/fimmu.2024.1330228

**Published:** 2024-04-12

**Authors:** Dong Kwon Kim, Chai Young Lee, Yu Jin Han, So Young Park, Heekyung Han, Kwangmin Na, Mi Hyun Kim, Seung Min Yang, Sujeong Baek, Youngtaek Kim, Joon Yeon Hwang, Seul Lee, Seong-san Kang, Min Hee Hong, Sun Min Lim, Jii Bum Lee, Jae Hwan Kim, Byoung Chul Cho, Kyoung-Ho Pyo

**Affiliations:** ^1^ Brain Korea 21 PLUS Project for Medical Science, Yonsei University College of Medicine, Seoul, Republic of Korea; ^2^ Severance Biomedical Science Institute, Yonsei University College of Medicine, Seoul, Republic of Korea; ^3^ Jeuk Institute for Cancer Research, Jeuk Co. Ltd., Gumi, Republic of Korea; ^4^ Division of Medical Oncology, Department of Internal Medicine and Yonsei Cancer Center, Severance Hospital, Yonsei University College of Medicine, Seoul, Republic of Korea; ^5^ Yonsei New Il Han Institute for Integrative Lung Cancer Research, Yonsei University College of Medicine, Seoul, Republic of Korea; ^6^ Department of Research Support, Yonsei Biomedical Research Institute, Yonsei University College of Medicine, Seoul, Republic of Korea

**Keywords:** aryl hydrocarbon receptor, tumor microenvironment, T-lymphocyte, macrophage, regulatory T cell

## Abstract

**Introduction:**

Aryl hydrocarbon receptor (AhR) is a transcription factor that performs various functions upon ligand activation. Several studies have explored the role of AhR expression in tumor progression and immune surveillance. Nevertheless, investigations on the distribution of AhR expression, specifically in cancer or immune cells in the tumor microenvironment (TME), remain limited. Examining the AhR expression and distribution in the TME is crucial for gaining insights into the mechanism of action of AhR-targeting anticancer agents and their potential as biomarkers.

**Methods:**

Here, we used multiplexed immunohistochemistry (mIHC) and image cytometry to investigate the AhR expression and distribution in 513 patient samples, of which 292 are patients with one of five solid cancer types. Additionally, we analyzed the nuclear and cytosolic distribution of AhR expression.

**Results:**

Our findings reveal that AhR expression was primarily localized in cancer cells, followed by stromal T cells and macrophages. Furthermore, we observed a positive correlation between the nuclear and cytosolic expression of AhR, indicating that the expression of AhR as a biomarker is independent of its localization. Interestingly, the expression patterns of AhR were categorized into three clusters based on the cancer type, with high AhR expression levels being found in regulatory T cells (Tregs) in non-small cell lung cancer (NSCLC).

**Discussion:**

These findings are anticipated to serve as pivotal evidence for the design of clinical trials and the analysis of the anticancer mechanisms of AhR-targeting therapies.

## 1 Introduction

Aryl hydrocarbon receptor (AhR) is a cytosolic ligand-activated transcription factor that is activated by various exogenous and endogenous molecules. Once activated, AhR translocates to the nucleus and forms a heterodimer with AhR nuclear translocator (ARNT). The AhR-ARNT complex then binds to specific DNA sequences known as xenobiotic response elements, which are located in the promoter region of a target gene, initiating the transcription of the downstream target genes (1, 2). AhR is widely expressed in the body and regulates the expression of various genes, thereby governing various cellular processes, including detoxification and metabolism (3–5). AhR can have different biological effects depending on its context, the cell type, and the activating ligand.

AhR plays an important role in carcinogenesis and tumor immunity (6–8). However, whether AhR positively or negatively regulates carcinogenesis remains inconclusive. DiNatale et al. demonstrated that AhR expression was higher in head and neck squamous cell carcinoma (HNSCC) tissues than in normal tissues, and AhR antagonists significantly decreased HNSCC cell migration (9). AhR has both tumor-promoting and suppressive effects in bladder cancer (10). AhR expression is higher in lung cancer tissues than in normal tissues, indicating that AhR has a tumor-suppressive role in carcinogenesis (11). In contrast, Breitenbucher et al. suggested that the knockdown of endogenous AhR in mice induced epithelial-mesenchymal transition signatures and metastasis (12).

Since AhR has been shown to be involved in carcinogenesis and tumor immunity, extensive studies have been conducted to elucidate the role of AhR in the tumor microenvironment (TME). Despite these efforts, the role of AhR in different cancer types at different stages remains unclear. The controversy surrounding the effect of AhR on carcinogenesis and tumor immunity may be attributed to the type, stage, and degree of cancer malignancy. Since AhR is an emerging potential target for anticancer therapy, clarifying its role in specific cancer types is crucial to facilitate patient stratification. Investigating the expression and localization of AhR may be a logical starting point since it may help unveil the molecular mechanisms involved in carcinogenesis.

Here, we evaluated the AhR expression and distribution in five solid cancer types: HNSCC, bladder cancer, colorectal cancer, esophageal cancer, and non-small cell lung cancer (NSCLC). Additionally, since AhR translocates from the cytosol to the nucleus upon activation, the comparison of the nuclear and cytosolic expression of AhR may provide valuable insights into the differential roles of AhR in each cancer type (13). To date, the number of AhR studies done on large cohorts is limited. By investigating a large cohort of patients with different clinical parameters, we aimed to address the knowledge gap regarding the expression of AhR in various cancers. Our findings may elucidate the mechanisms underlying AhR-mediated pathways in cancer and immune cells in the TME across different cancer types. We hope that our findings will pave the way for the development of anticancer therapies targeting AhR and for patient stratification to optimize the treatment approaches.

## 2 Methods

### 2.1 Sample preparation

Five tissue microarray (TMA) slides were purchased from Tissue Array. These slides were used to analyze the tissue samples from patients with different cancers: bladder cancer (Tissue ID: BL481d, 48 cores), colorectal cancer (Tissue ID: CO1002d, 100 cores), HNSCC (Tissue ID: HN802d, 80 cores), NSCLC (Tissue ID: LC10012b, 100 cores), and esophageal cancer (Tissue ID: ES1922a, 192 cores). Each TMA core had a specific size (2.0 mm for bladder cancer, 1.0 mm for colorectal cancer, 1.5 mm for HNSCC, 1.0 mm for NSCLC, and 1.0 mm for esophageal cancer). The clinical and demographic parameters of the patients are presented in **Table 1**. Out of a total of 520 cores, three cores from colorectal cancer, two cores from NSCLC, and two cores from esophageal cancer were excluded from the analysis due to their absence on the slides during the experimental evaluation. 

**Table 1:** Demographic and clinical information of the patient samples from TMA slides of five solid cancer types, including age, sex, cancer grade, cancer stage, and metastasis.

Bladder			Colon			Esophagus			Head and neck			Lung		
Age	Total Number (n)	Percentage (%)	Age	Total Number (n)	Percentage (%)	Age	Total Number (n)	Percentage (%)	Age	Total Number (n)	Percentage (%)	Age	Total Number (n)	Percentage (%)
11-20	1	2.1	11-20	–	–	11-20	–	–	11-20	2	2.5	11-20	2	2
21-30	2	4.2	21-30	8	7.9	21-30	–	–	21-30	7	8.8	21-30	4	4
31-40	5	10.4	31-40	15	14.9	31-40	–	–	31-40	5	6.3	31-40	8	8
41-50	11	22.9	41-50	22	21.8	41-50	23	11.9	41-50	24	30	41-50	8	8
51-60	13	27.1	51-60	32	31.7	51-60	62	32.1	51-60	19	23.8	51-60	34	34
61-70	9	18.8	61-70	14	13.9	61-70	92	47.7	61-70	17	21.3	61-70	38	38
71-80	5	10.4	71-80	10	9.9	71-80	16	8.3	71-80	6	7.5	71-80	6	6
81-90	2	4.2	81-90	–	–	81-90	–	–	81-90	–	–	81-90	–	–
Sex	Total Number (n)	Percentage (%)	Sex	Total Number (n)	Percentage (%)	Sex	Total Number (n)	Percentage (%)	Sex	Total Number (n)	Percentage (%)	Sex	Total Number (n)	Percentage (%)
Female	13	27.1	Female	23	22.8	Female	40	20.7	Female	12	15	Female	18	18
Male	35	72.9	Male	78	77.2	Male	153	79.3	Male	68	85	Male	82	82
Grade	Total Number (n)	Percentage (%)	Grade	Total Number (n)	Percentage (%)	Grade	Total Number (n)	Percentage (%)	Grade	Total Number (n)	Percentage (%)	Grade	Total Number (n)	Percentage (%)
Unknown	–	–	Unknown	57	56.4	Unknown	97	50.3	Unknown	20	25	Unknown	58	58
1	–	–	1	12	11.9	1	30	15.5	1	16	20	1	3	3
1-2	–	–	1-2	–	–	1-2	–	–	1-2	1	1.3	1-2	–	–
2	–	–	2	25	24.8	2	34	17.6	2	33	41.3	2	18	18
2-3	–	–	2-3	–	–	2-3	–	–	2-3	–	–	2-3	2	2
3	–	–	3	7	6.9	3	32	16.6	3	10	12.5	3	19	19
Stage	Total Number (n)	Percentage (%)	Stage	Total Number (n)	Percentage (%)	Stage	Total Number (n)	Percentage (%)	Stage	Total Number (n)	Percentage (%)	Stage	Total Number (n)	Percentage (%)
Unknown	8	16.7	Unknown	64	63.4	Unknown	–	–	Unknown	10	12.5	Unknown	55	55
I	9	18.8	I	–	–	I	–	–	I	3	3.8	I	–	–
IA	–	–	IA	–	–	IA	–	–	IA	–	–	IA	1	1
IB	–	–	IB	–	–	IB	–	–	IB	–	–	IB	27	27
II	15	31.3	II	–	–	II	–	–	II	21	26.3	II	–	–
IIA			IIA	24	23.8	IIA	–	–	IIA	–	–	IIA	12	12
III	15	31.3	III	–	–	III	–	–	III	21	26.3	III	3	3
IIIB	–	–	IIIB	10	9.9	IIIB	–	–	IIIB	–	–	IIIB	2	2
IIIC	–	–	IIIC	2	2.0	IIIC	–	–	IIIC	–	–	IIIC	–	–
IV	1	2.1	IV	–	–	IV	–	–	IV	–	–	IV	–	–
IVA	–	–	IVA	–	–	IVA	–	–	IVA	25	31.3	IVA	–	–
Type	Total Number (n)	Percentage (%)	Type	Total Number (n)	Percentage (%)	Type	Total Number (n)	Percentage (%)	Type	Total Number (n)	Percentage (%)	Type	Total Number (n)	Percentage (%)
AT	–	–	AT	14	13.9	AT	71	36.8	AT	–	–	AT	45	45
Malignant	40	83.3	Malignant	37	36.6	Malignant	97	50.3	Malignant	70	87.5	Malignant	45	45
Metastasis	–	–	Metastasis	9	8.9	Metastasis	–	–	Metastasis	–	–	Metastasis	–	–
NAT	–	–	NAT	22	21.8	NAT	25	13.0	NAT	–	–	NAT	–	–
Normal	8	16.7	Normal	19	18.8	Normal	–	–	Normal	10	12.5	Normal	10	10

### 2.2 Multiplexed immunohistochemistry (mIHC)

For the multiplexed immunohistochemical staining of the sections, we used BOND RX Fully Automated Research Stainer (cat: 21.2821; Leica Biosystems) and an Opal Polaris 7Color IHC Detection Kit (cat: P-000003, Akoya Biosciences). All procedures were performed according to the manufacturer’s instructions. In summary, deparaffinized sections were incubated with citrate- or Tris-based antigen unmasking solutions (for heat-induced epitope retrieval) at 98°C for 20 min. They were then treated with hydrogen peroxide and a protein-blocking reagent to prevent the nonspecific binding of antibodies to the sections. Sections were sequentially treated with the primary antibodies, horseradish peroxidase (HRP)-conjugated antibodies, and specific fluorophores to detect the proteins of interest. Multiple staining rounds were performed using the following anti-human antibodies: anti-AhR (cat: LS-C783005-100; LS Biosciences), anti-CD68 (cat: 76437; Cell Signaling Technology), anti-CD4 (cat: ab181724; Abcam), anti-CD8 (cat: CD8-4B11-L-CE; Leica Biosystems), anti-FoxP3 (cat: 98377; Cell Signaling Technology, anti-PanCK (cat: AE1/AE3-601-L-CE; Leica Biosystems). Tissue sections were counterstained with Spectral DAPI (4′,6-diamidino-2-phenylindole, cat: SKU FP1490; Akoya Biosciences).

### 2.3 Whole-slide scanning and matrix data generation

Images (200× magnification) of the whole tissue contents were generated using whole-slide scanning by using Vectra Polaris Automated Quantitative Pathology Imaging System (cat: CLS143455; Akoya Biosciences). Multispectral images for analysis were defined and selected within the whole tissue using Phenochart whole slide contextual viewer software (version 1.12 for Windows; Akoya Biosciences). The inForm^®^ software (version 2.6 for Windows; Akoya Biosciences), equipped with an integrated algorithm for tissue analysis, was employed to transform the multispectral image data into numerical data. These data encompassed both numerical and spatial information regarding the tumor nest and stromal region, defining the cell components (nuclear, cytosolic, and membrane margins), classifying the cell populations and the intensities of each marker. Cell populations were discerned based on the distinctive patterns of CD marker expression, each exhibiting unique cellular properties, such as nuclear, cytosolic, and membranal sizes and shapes (CD4/8 = T cells; CD68 = macrophages; Pan-CK = epithelial cells or cancer cells; FoxP3 = Treg). The differentiation between the tumor nest and stroma, as well as the area calculations, were done based on the pan-cytokeratin staining patterns by utilizing an algorithm integrated into the inForm^®^ software. The integrated matrix files were derived from the segmentation of cells and tissues from the TMA data.

### 2.4 Image cytometry and analysis

The required fluorescence values and labels were extracted from a previously obtained matrix file obtained using inForm^®^. These data included the fluorescence intensity in the nucleus and cytosol. The extracted CSV file was converted into an FCS file using the R program and the packages BioBase (R/Bioconductor, 3.17), FlowCore (R/Bioconductor, 2.10), and FlowViz (R/Bioconductor, 3.17). The FCS file was subsequently converted into a raw file for cytometric image analysis using FlowJo (version 10.9.0 for Windows). Through this process, we analyzed the cancer and immune cells within the TME and quantified the AhR expression levels (**Supplementary Figure 1**). The image cytometry data obtained were processed via gating to determine the proportion of cells expressing AhR by distinguishing them based on the T-cell markers CD4/8, the macrophage marker CD68, the cancer marker Pan-CK, and the Treg marker FoxP3 (**Supplementary Figure 2**). The image cytometry results obtained, which were distinguished based on the nuclear and cytosolic AhR expression, were used for analysis. A complex heatmap was generated using pheatmap (R/CRAN, 1.0.12), and the bar plots were visualized using ggplot2 (R/CRAN, 3.4.3) to generate the outcomes.

### 2.5 Statistical analysis

Data are reported as the mean ± SEM. Statistical analysis was performed using analysis of variance (ANOVA) with the Mantel-Cox log-rank test to assess the significant differences using GraphPad Prism (version 7.00 for Windows; GraphPad Software). Statistical analysis of the flow cytometry data was performed using the t-test in GraphPad Prism. The correlations between the nuclear and cytosolic AhR expression values between normal and malignant tissues were analyzed using the chi-square test with χ2 correction or Fisher’s exact test for the categorical variables.

## 3 Results

### 3.1 Multiplexed immunohistochemistry (mIHC) analysis reveals differential AhR expression in the tumor microenvironment of five cancer types

**Figure 1:**
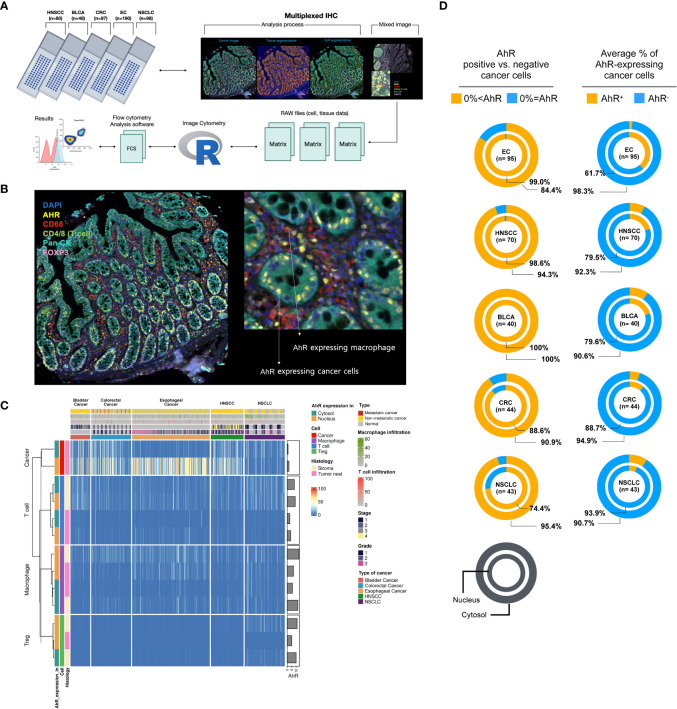
AhR expression and localization in the TME of bladder, colorectal, esophageal, head and neck, and non-small cell lung cancers. **(A)** Scheme of the workflow used for generating AhR expression data from five FFPE TMA slides: NSCLC (non-small cell lung cancer, N = 98), HNSCC (head and neck squamous cell carcinoma, N = 80), CRC (colorectal cancer, N = 97), EC (esophageal cancer, N = 190), and BLCA (bladder cancer, N = 48). Prior to cell segmentation and tissue segmentation using the deep-learning inForm algorithm, five TMA slides were stained with six antibodies, DAPI, and anti-AhR, -CD68, -CD4, -CD8, -Pan-CK, and -FoxP3, using Vectra Polaris for mIHC image generation. The resulting data were manipulated to perform image cytometry for phenotyping and AhR expression analysis. **(B)** Representative mIHC image of the region of interest stained with six antibodies: DAPI for nucleus, Pan-CK for cancer/epithelial cells, CD4 and CD8 for T cells, CD68 for macrophages, and FoxP3 for Tregs. **(C)** Heatmap summarizing the AhR expression in five different tissue samples: NSCLC, HNSCC, colorectal cancer, esophageal cancer, and bladder cancer. The resulting data were clustered based on the AhR expression in the cytosol or nucleus of cancer/epithelial cells, T cells, macrophages, and Tregs. **(D)** Pie charts depicting the AhR-positive (AhR^+^) and -negative (AhR^-^) cancer cells (left) and average percentage of AhR-expressing cancer cells (right) in the cytosol and nucleus of NSCLC, HNSCC, CRC (colorectal cancer), EC (esophageal cancer), and BLCA (bladder cancer) cells.

To investigate AhR expression in the TME of the five cancer types (HNSCC, bladder cancer, colorectal cancer, esophageal cancer, and NSCLC), which contains cancer and immune cells, five TMA slides were subjected to mIHC analysis. The tissue samples from 520 healthy and malignant patients were successfully stained for cell phenotyping and analysis of the heterogeneity of AhR expression. **Figure 1A** illustrates the process flow used for image cytometry of the slides used for TMA. After performing mIHC staining, the TMA slides underwent whole-slide imaging to analyze the various fluorescence signals and quantify the relevant information from the discrete tumor nests and stromal regions identified, as well as to determine the expression of individual fluorescent markers in each cell within the TME. The acquired data were converted into a CSV matrix file and further processed into input files compatible with the image cytometry analysis ones, FCS files, for the subsequent analysis. The overall workflow of this analytical method is described in **Supplementary Figure 1**, and the gating method used for the image cytometry data is detailed in **Supplementary Figure 2**. **Figure 1B** shows a representative mIHC image of the cancer-adjacent colon tissue stained with six markers: DAPI for the nucleus, Pan-CK for cancer or epithelial cells, CD4 and CD8 for T cells, CD68 for macrophages, and FoxP3 for Tregs. AhR-expressing epithelial cells and macrophages are represented in yellow and are surrounded by cyan blue Pan-CK+ epithelial cells and red CD68+ macrophages, respectively, as indicated by the arrows (**Figure 1B**, image on the right). The columns in the heatmap in **Figure 1C** include clinical metadata such as the stage, grade, and metastasis, as well as information regarding T-cell and tumor-associated macrophage (TAM) infiltration. In the rows, the AhR expression levels in the nucleus and cytosol of each immune cell are presented as percentages. Additionally, the positional information of the cells, whether in the tumor nest or stroma, is provided in the rows. The key color in the heatmap indicates the proportion of cells expressing AhR among all cells constituting the tissue core. For example, 100% of nucleus AhR+ Pan-CK means that 100% of the cancer cells express AhR in the nucleus. Based on the dendrogram of each marker obtained through hierarchical clustering (H-clustering), the AhR expression pattern was distinct between cancer and immune cells, with clear AhR expression patterns being observed within T cells, Tregs, and macrophages. In most cases, the highest AhR expression level was observed in the nucleus, and immune cells tended to have a higher AhR expression in the stroma than in the tumor nest. The clinical metadata and expression patterns for each core are shown in **Supplementary Tables 1**–**5**. AhR expression was the most pronounced in tumors. AhR expression was predominantly observed across the five cancer types, even when distinguishing between the nucleus and cytosol. In particular, for esophageal cancer, a 99% and 84.4% AhR expression was observed in the nucleus and cytosol, respectively, with the highest proportion of AhR non-expression being found in the cytosol (n = 95). Interestingly, bladder cancer exhibited a 100% AhR expression in both the nucleus and cytosol (n = 40). In colorectal cancer, AhR expression in the nucleus and cytosol was found in 88.6% and 90.9% of samples (n = 40), respectively. In NSCLC, nuclear and cytosolic AhR expression was observed in 74.4% and 95.4% of the samples (n = 43), with the highest proportion of non-expression being found in the nucleus (**Figure 1D**, left image). To further investigate this trend, we assessed the average AhR expression in malignant samples across five different cancer types. Notably, the average AhR expression does not indicate the percentage of individual cancer tissues expressing AhR. Instead, it signifies the average proportion of cells expressing AhR in the TME. The highest nuclear AhR expression (38.3%, n = 95) was found in esophageal cancer samples. Interestingly, in contrast with the nuclear expression proportion, the cytosolic expression was the lowest (1.7%) among all the cancer samples. In HNSCC, the nuclear and cytosolic AhR expression was 20.5% and 7.7%, respectively (n = 70). Similarly, bladder cancer demonstrated a comparable AhR expression pattern, with expression rates of 20.4% and 9.4%, respectively. In colorectal cancer, the nuclear and cytosolic AhR expression levels were 11.3% and 5.1% (n = 44), whereas in NSCLC, they were 6.1% and 9.3% (n = 43), respectively. Notably, NSCLC cells exhibited a higher AhR expression in the cytosol than in the nucleus, distinguishing it from other cancer types (**Figure 1D**, image on the right). Chi-square analysis was conducted to compare the nuclear and cytosolic AhR expression between normal and malignant tissues. Overall, no significant difference in AhR expression was observed between normal and malignant tissues across the three cancer types analyzed (colorectal cancer, esophageal cancer, and NSCLC). Bladder cancer and HNSCC were excluded from the chi-square analysis because samples lacking AhR characteristics (AhR expression = 0%) were absent in both cancer types (**Supplementary Table 6**).

### 3.2 AhR expression patterns in head and neck squamous cell carcinoma (HNSCC)

**Figure 2:**
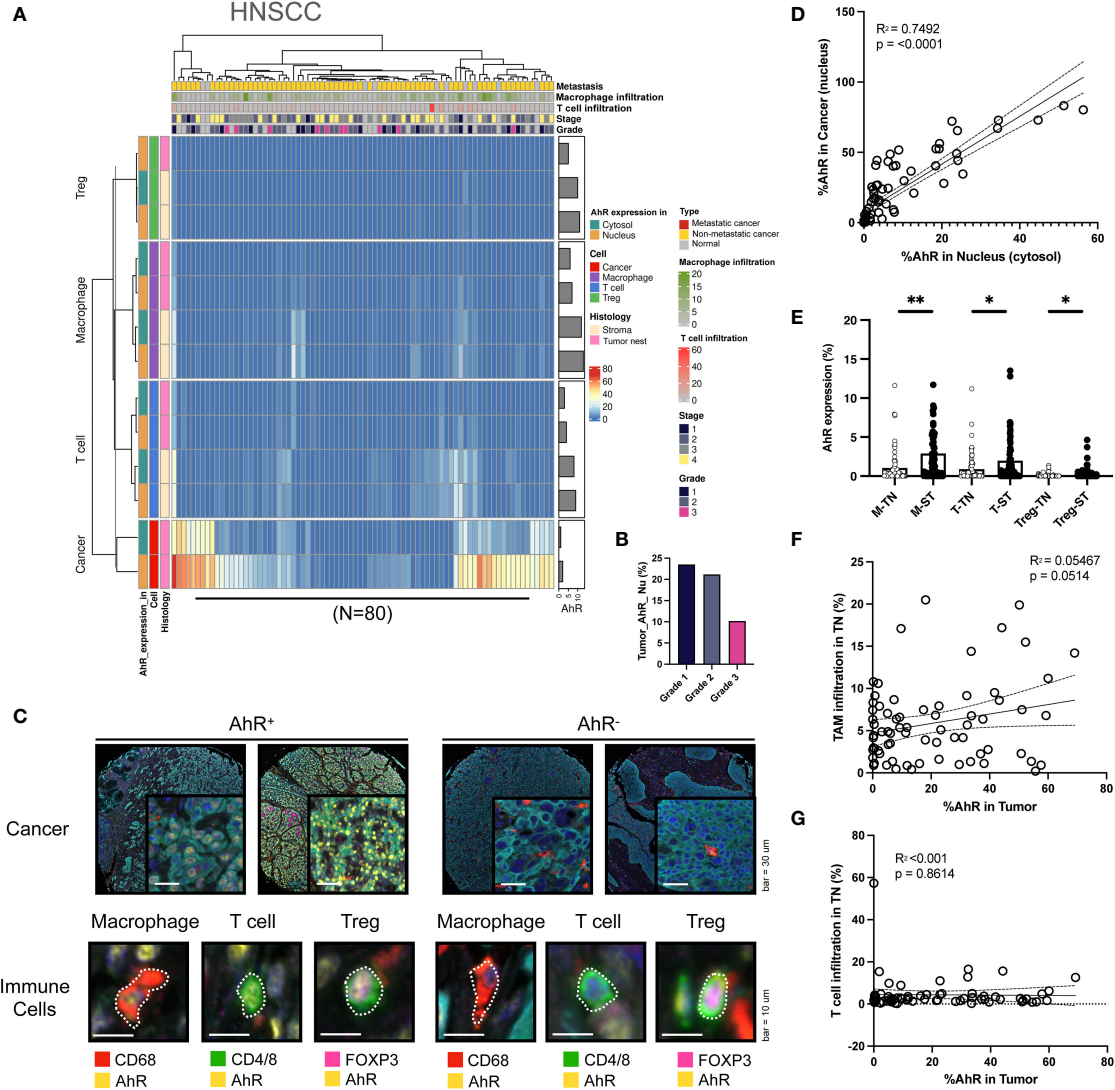
AhR expression patterns in head and neck squamous cell carcinoma (HNSCC). **(A)** Heatmap summarizing the AhR expression and localization in cancer/epithelial cells, T cells, macrophages, and Tregs in normal and malignant head and neck tissues (N = 80). **(B)** AhR expression in cancer cells in malignant head and neck tissues with varying cancer grades. **(C)** Representative images of AhR-expressing (AhR^+^) and non-AhR-expressing (AhR^-^) head and neck cancer cells, along with AhR-expressing immune cells, including macrophages, T cells, and Tregs. **(D)** AhR expression in the cytosol was positively correlated with that in the nucleus (*P* < 0.0001). **(E)** AhR expression in T cells (T), macrophages (M), and Tregs in the stroma (ST) and tumor nest (TN). **(F)** Correlation plot showing no significant correlation between AhR expression in the cancer cell nucleus and the TAM infiltration degree in the tumor nest (TN)(*P* = 0.0514). **(G)** Correlation plot showing no significant correlation between AhR expression in the cancer cell nucleus and the T-cell infiltration degree in the TN. *P < 0.05; **P < 0.01.

To comprehensively analyze the AhR expression patterns in individual cancer types, heat maps were created for each specific cancer type and analyzed. The clusters in the heatmap shown in **Figure 2A** were divided based on the nuclear or cytosolic AhR expression in cancer and immune cells in the head and neck tissue samples. Upon examination of both normal and malignant head and neck tissue samples, H-clustering based on AhR expression revealed that the highest AhR expression was found in these cancer cells, particularly in the nucleus, as opposed to the cytosol. Although AhR was not detected in immune cells within the tumor nest region, AhR was expressed in T cells and macrophages in the stromal regions (**Figure 2A**). The influence of the different cancer stages on AhR expression in HNSCC cells was further investigated, and a diminishing trend in AhR expression with an advancing cancer grade was observed (**Figure 2B**). Representative mIHC images of AhR-expressing cancer cells and immune cells (macrophages, T cells, and Tregs) in malignant head and neck tissues are found in **Figure 2C** (left side). AhR expression in cancer cells, localized to the nucleus, was characterized by distinct yellow staining in the DAPI regions of each cancer cell line, as identified via cyan blue Pan-CK staining. A diffuse expression, with a lower yellow staining intensity surrounding the nucleus, illustrates the cytosolic AhR expression. Macrophages were identified by the red CD68 staining of the cell body. Overlapping blue DAPI and yellow AhR staining indicated AhR expression. T-cell AhR expression is represented by yellow staining in the nucleus and cell body region and phenotyped by green CD4/8 staining. To identify the Tregs, FoxP3, which stains the nucleus of T cells, was used. Tregs were identified by a magenta FoxP3 stained nucleus surrounded by a green CD4/8 stained cell body region. AhR expression in Tregs is illustrated by the overlap of magenta FoxP3 and yellow AhR staining. On the right side of **Figure 2C**, the five images demonstrate the absence of AhR expression in both cancer and immune cells. These cells lack yellow staining in both the nucleus and cytosol (**Figure 2C**). To investigate the correlation between AhR expression in the nucleus and cytosol, the percentage of cells expressing AhR in each compartment in individual head and neck samples was examined. The analysis revealed a statistically significant positive correlation between the parameters, indicating a positive relationship between AhR expression in the nucleus and cytosol (P < 0.0001) (**Figure 2D**). **Figure 2E** reveals a strong positive relationship between AhR expression in individual immune cell types in the tumor nest and that in the stroma (**Figure 2E**). The potential effect of immune cell infiltration into the tumor nest on AhR expression in malignant tissues was investigated. Although not statistically significant, a positive correlation was observed between the TAM infiltration degree and AhR expression in HNSCC cells (P = 0.0514) (**Figure 2F**). In contrast, no discernible correlation was observed between the T-cell infiltration degree in the tumor nest and AhR expression in malignant tissues (**Figure 2G**).

### 3.3 AhR expression patterns in bladder cancer

**Figure 3:**
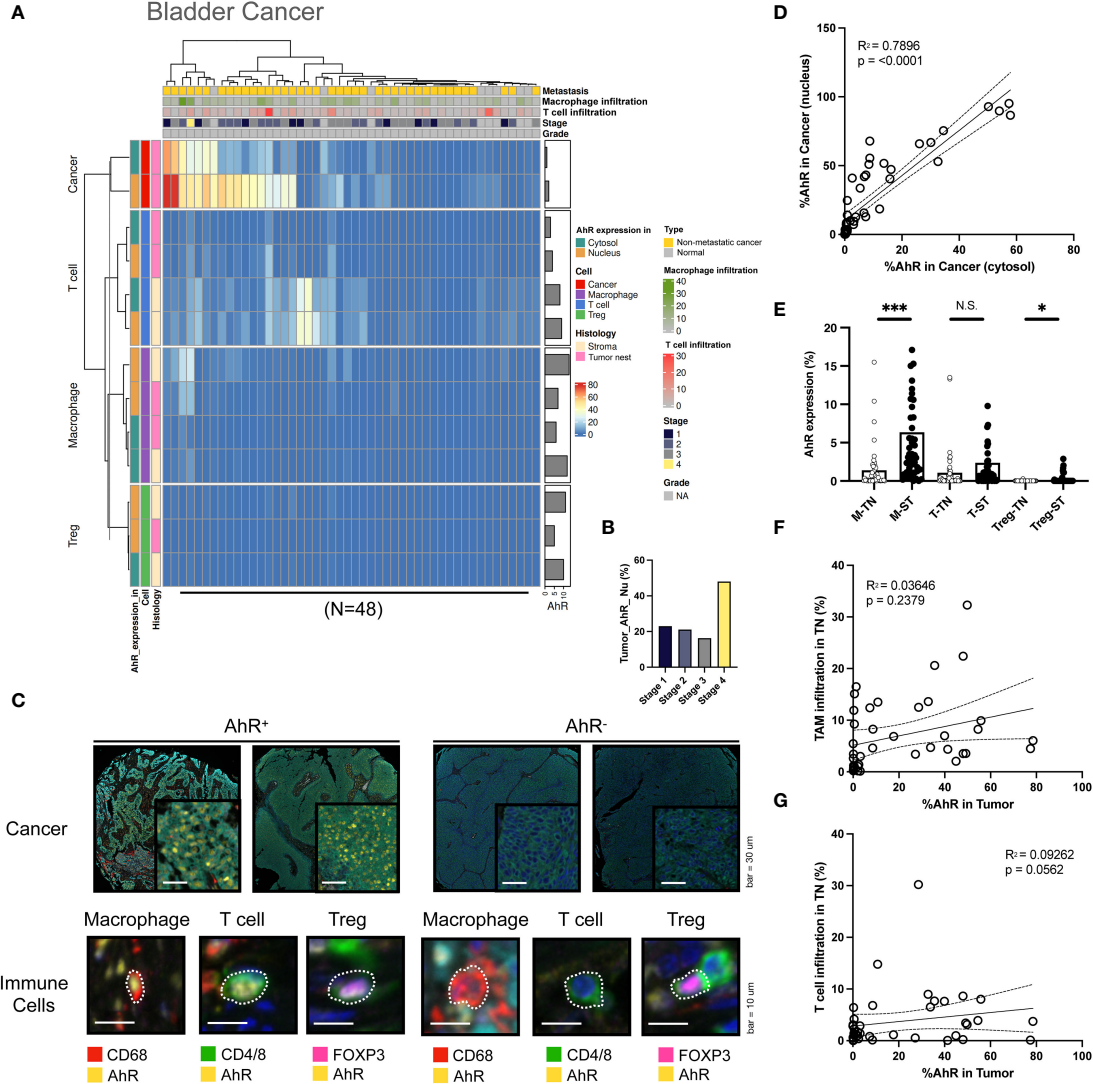
AhR expression patterns in bladder cancer. **(A)** Heatmap summarizing the AhR expression and localization in cancer/epithelial cells, T cells, macrophages, and Tregs in normal and malignant bladder tissues (N = 48). **(B)** AhR expression in malignant bladder tissues of varying cancer stages. **(C)** Representative images of AhR-expressing (AhR^+^) and non-AhR^-^expressing (AhR^-^) bladder cancer cells, along with AhR-expressing immune cells, including macrophages, T cells, and Tregs. **(D)** AhR expression in the cytosol is positively correlated with the nuclear AhR expression (*P* < 0.0001). **(E)** AhR expression in T cells (T), macrophages (M), and Tregs in the stroma (ST) and tumor nest (TN). **(F)** Correlation plot showing no significant correlation between AhR expression in cancer cell nucleus and the TAM infiltration degree in the TN(*P* = 0.0562). **(G)** Correlation plot showing no significant correlation between the AhR expression in cancer cell nucleus and the T-cell infiltration degree in the TN. N.S., no significant difference, *P < 0.05, ***P < 0.001.

AhR expression was high in bladder cancer cells, similar to the results obtained in HNSCC cells. Similar to HNSCC, the AhR expression was higher in the nucleus than in the cytosol. Additionally, AhR was detected in T cells and macrophages in the stromal regions (**Figure 3A**). In contrast with HNSCC, which shows a AhR expression decrease with an increasing cancer grade, AhR expression was consistent across grade 1–3 cancers, whereas it increased in the stage 4 bladder cancer samples (**Figure 3B**). The images on the left in **Figure 3C** illustrate representative mIHC images of AhR-expressing cancer and immune cells (macrophages, T cells, and Tregs), which can be distinguished by the yellow AhR stained areas in the bladder cancer tissues. In contrast, the cancer and immune cells in the right images exhibit no AhR expression, as indicated by the absence of yellow regions. Similar to what was found for HNSCC, the nuclear AhR expression was significantly positively correlated with the cytosolic one (P < 0.0001) (**Figure 3D**). Similar to those in HNSCC, the AhR expression levels in specific immune cell types (macrophages, T cells, and Tregs) in the tumor nest were positively correlated with the stromal ones (**Figure 3E**). No clear relationship between the tumor-associated macrophages (TAM) infiltration level in the tumor nest and AhR expression was observed in cancer cells (**Figure 3F**). Nevertheless, the T-cell infiltration level in the tumor nest was positively correlated with AhR expression in cancer cells (**Figure 3G**).

### 3.4 AhR expression patterns in colorectal cancer

**Figure 4:**
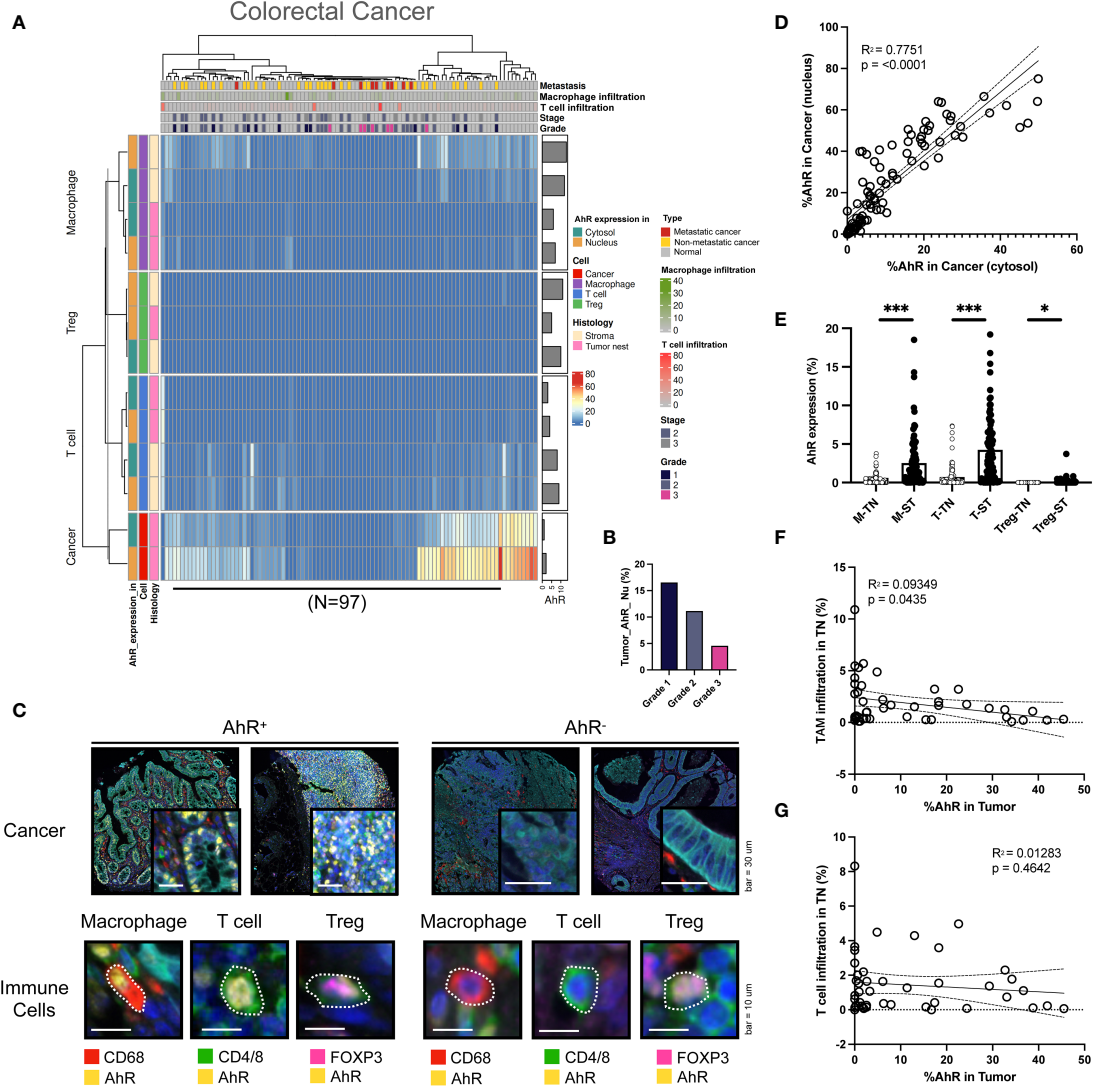
AhR expression patterns in colorectal cancer. **(A)** Heatmap summarizing the AhR expression and localization in cancer/epithelial cells, T cells, macrophages, and Tregs in normal and malignant colorectal tissues (N = 97). **(B)** AhR expression in malignant colorectal tissues of varying cancer grades. **(C)** Representative images of AhR-expressing (AhR^+^) and non-AhR-expressing (AhR^-^) colorectal cancer cells, along with AhR-expressing immune cells, including macrophages, T cells, and Tregs. **(D)** AhR expression in the cytosol is positively correlated with the nuclear AhR expression (*P* < 0.0001). **(E)** AhR expression in T cells (T), macrophages (M), and Tregs in the stroma (ST) and tumor nest (TN). **(F)** Correlation plot showing an increase in AhR expression in cancer cell nucleus as the degree of TAM infiltration in the TN decreased. **(G)** Correlation plot showing no significant correlation between AhR expression in cancer cell nucleus and the T-cell infiltration degree in the TN. *P < 0.05, ***P < 0.001.

In colorectal cancer cells, the nuclear AhR expression was higher than the cytosolic one. AhR was also detected T cells and macrophages in the stroma (**Figure 4A**). The AhR expression in cancer cells increased with an increasing colorectal cancer grade, similar to the results obtained for HNSCC (**Figure 4B**). In **Figure 4C**, the images on the left show mIHC images of AhR-expressing cancer cells and immune cells (macrophages, T cells, and Tregs) in colorectal tissues, which are stained in yellow. In contrast, the images on the right depict cancer and immune cells lacking AhR expression, as evidenced by the absence of yellow staining (**Figure 4C**). Similar to the results obtained for the other cancer types, the nuclear and cytosolic AhR expression were significantly positively correlated in colorectal cancer (**Figure 4D**). The AhR expression levels in each immune cell type (macrophages, T cells, and Tregs) within the tumor nest were positively correlated with the stromal AhR expression in the corresponding cell type, which was comparable to that found in bladder cancer and HNSCC (**Figure 4E**). The immune cell infiltration degree in the tumor nest was not correlated with AhR expression in the tumor region for either TAMs or T cells (**Figures 4F, G**).

### 3.5 AhR expression patterns in esophageal cancer

**Figure 5:**
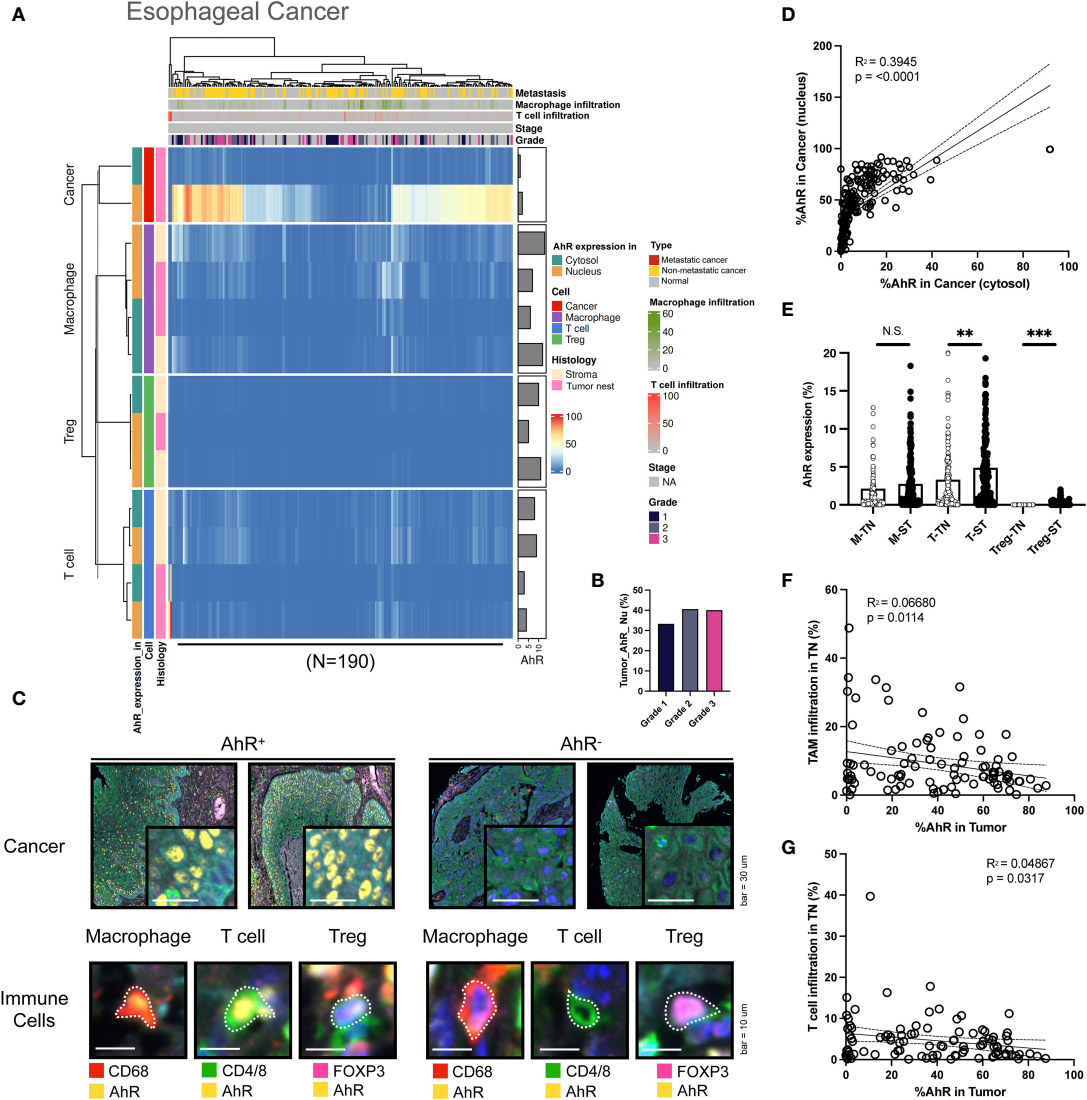
AhR expression patterns in esophageal cancer. **(A)** Heatmap summarizing the AhR expression and localization in cancer/epithelial cells, T cells, macrophages, and Tregs in normal and malignant esophageal tissues (N = 190). **(B)** AhR expression in malignant esophageal tissues of varying cancer grades. **(C)** Representative images of AhR-expressing (AhR^+^) and non-AhR-expressing (AhR^-^) esophageal cancer cells, along with AhR-expressing immune cells, including macrophages, T cells, and Tregs. **(D)** AhR expression in the cytosol is positively correlated with the nuclear AhR expression (*P* < 0.0001). **(E)** AhR expression in T cells (T), macrophages (M), and Tregs in the stroma (ST) and tumor nest (TN). **(F)** Correlation plot showing no significant correlation between AhR expression in cancer cell nucleus and the TAM infiltration degree in the TN. **(G)** Correlation plot showing no significant correlation between AhR expression in cancer cell nucleus and the T-cell infiltration degree in the TN. N.S., no significant difference, **P < 0.01, ***P < 0.001.

Similar to the aforementioned cancer types, AhR is abundantly expressed in esophageal cancer cells. AhR expression was higher in the nucleus than in the cytosol. Compared with that in bladder cancer and HNSCC, the nuclear AhR expression in cancer cells was significantly higher than the cytosolic one. Moreover, AhR-expressing T cells and macrophages were not only observed in the stromal regions, but also within the tumor nest, indicating that AhR is expressed in the immune cells that infiltrated the tumor nest. This pattern was not observed in other cancer types (**Figure 5A**). In contrast with that obtained for the aforementioned cancer types, the nuclear AhR expression in cancer cells increased with an advancing cancer grade (**Figure 5B**). The images on the left in **Figure 5C** show mIHC images of AhR-expressing cancer and immune cells (macrophages, T cells, and Tregs) in esophageal tissues, stained yellow. Conversely, the images on the right depict cancer and immune cells that do not express AhR, as evidenced by the absence of yellow staining. Compared with the other cancer types, the nuclear and cytosolic AhR expressions were significantly positively correlated in esophageal cancer (**Figure 5D**). The AhR expression levels in each immune cell type (macrophages, T cells, and Tregs) within the tumor nest were positively correlated with the stromal AhR expression in the corresponding cell type, a pattern consistent with those found for the other cancer types (**Figure 5E**). No clear pattern was found between the immune cell infiltration degree in the tumor nest and AhR expression in the tumor region for either TAMs or T cells (**Figures 5F, G**).

### 3.6 AhR expression patterns in non-small cell lung cancer (NSCLC)

**Figure 6:**
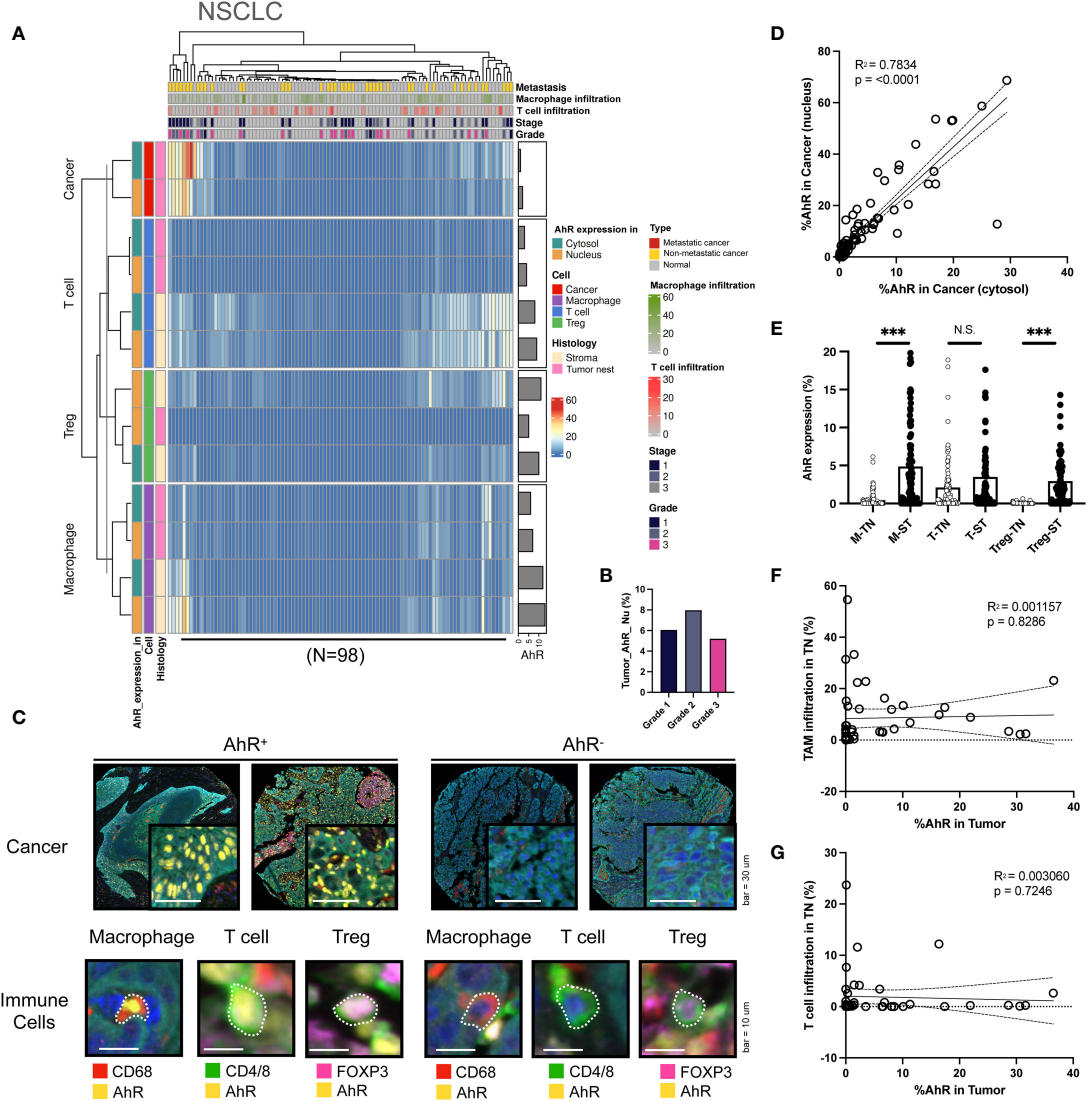
AhR expression patterns in non-small cell lung cancer (NSCLC). **(A)** Heatmap summarizing the AhR expression and localization in cancer/epithelial cells, T cells, macrophages, and Tregs in normal and malignant lung tissues (N = 98). **(B)** AhR expression in malignant NSCLC tissues of varying cancer grades. **(C)** Representative images of AhR-expressing (AhR^+^) and non-AhR-expressing (AhR^-^) lung cancer cells, along with AhR-expressing immune cells, including macrophages, T cells, and Tregs. **(D)** The AhR expression in the cytosol is positively correlated with the nuclear AhR expression (*P* < 0.0001). **(E)** AhR expression in T cells (T), macrophages (M), and Tregs in the stroma (ST) and tumor nest (TN). **(F)** Correlation plot showing no significant correlation between AhR expression in cancer cell nucleus and the TAM infiltration degree in the TN. **(G)** Correlation plot showing no significant correlation between AhR expression in cancer cell nucleus and the T-cell infiltration degree in the TN. N.S., no significant difference, ***P < 0.001.

Although AhR expression was elevated in NSCLC cells, similar to what was found in other cancer types, it was significantly higher in the cytosol than in the nucleus. Furthermore, T cells and macrophages in the stromal environment expressed AhR, consistent with the results obtained for other cancer types. AhR-expressing macrophages were not solely confined to the stroma, but were also identified within the tumor nest, representing a distinct AhR expression pattern in TAMs. Notably, AhR expression was found in Tregs within the stromal regions in both normal and malignant samples, a pattern that was not observed in the remaining four cancer types examined here (**Figure 6A**). No clear relationship between AhR expression and the NSCLC grade was found (**Figure 6B**). The images on the left in **Figure 5C** show representative mIHC images of AhR-expressing cancer and immune cells (macrophages, T cells, and Tregs), which are distinguished by the yellow AhR staining in lung tissues. In contrast, the cancer and immune cells in the images on the right exhibit no AhR expression, as indicated by the absence of yellow (**Figure 6C**). To investigate the correlation between the nuclear and cytosolic AhR expression, the percentage of cells expressing AhR in the nucleus and cytosol in individual bladder samples was examined. The analysis revealed a significantly positive correlation between these parameters, indicating a positive correlation between the nuclear and cytosolic AhR expression (**Figure 6D**). The AhR expression levels in each immune cell type (macrophages, T cells, and Tregs) within the tumor nest were positively correlated with the stromal AhR expression in the corresponding cell type, consistent with the findings observed in similar cancer types (**Figure 6E**). Again, no discernible correlation was observed between the extent of immune cell infiltration in the tumor nest and AhR expression in the tumor region for either TAMs or T cells (**Figures 6F, G**).

### 3.7 Differential AhR expression based on the cell type and location affects cancer characteristics

**Figure 7:**
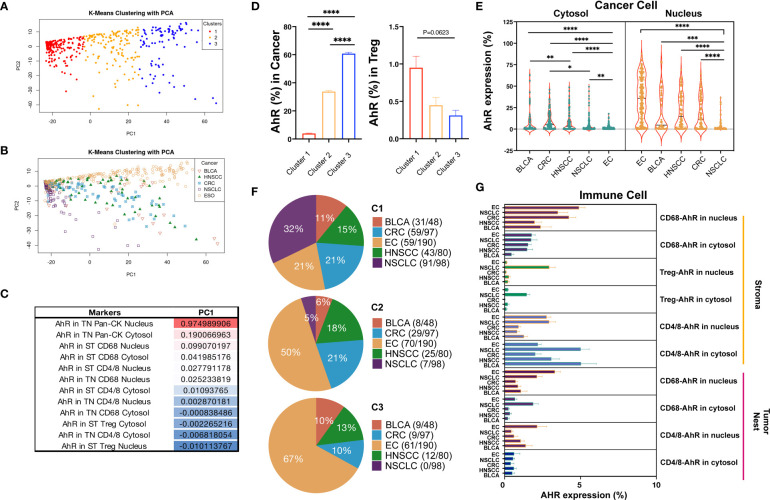
Differential AhR expression based on different cell types and locations. **(A)** PCA plot distinguishing the three clusters from each group. **(B)** PCA plot distinguishing the five cancer types from each group. **(C)** PC1 table displaying the AhR expression in different locations, including the cytosol vs. nucleus, tumor nest (TN) vs. stroma (ST), and different markers (Pan-CK, CD68, CD4/8, and Treg). **(D)** Bar graph showing the AhR expression in cancer cell nucleus and stromal Treg nucleus in three different clusters. **(E)** Pie chart representing the distribution of the five cancer types within the three different clusters. **(F)** Violin plot portraying the AhR expression in the cytosol vs. nucleus in the five cancer types. **(G)** Bar graph depicting the AhR expression in the tumor and immune cell types in the five cancer types. *P < 0.05, **P < 0.01, ***P < 0.001, ****P < 0.0001.

To thoroughly compare the five cancer types based on AhR expression, we applied a k-means clustering algorithm. K-means clustering and PCA were applied to individual tumor cores, each containing 13 features representing the AhR expression patterns, to identify meaningful clusters with significant components (P < 0.05; silhouette width = 0.45; **Supplementary Figure 3**). These clusters were distinguished along PC1 in the PCA plot, indicating a clear separation within each group (**Figures 7A, B**). Specifically, we focused on the analysis of cancer cells and macrophages situated at the extremes of PC1 and Tregs located at opposite ends (**Figure 7C**). Cluster 1 displayed the highest AhR expression in the cancer nucleus, whereas cluster 3 exhibited the lowest AhR expression in the cancer nucleus. AhR expression in the stromal macrophage nuclei was the most pronounced in cluster 2 (**Figure 7D**). This suggests that each cluster possesses distinct characteristics depending on the AhR expression and location. Consequently, we generated pie charts to explore the distribution of the five cancer types within each cluster (**Figure 7E**). In cluster 1, NSCLC was entirely absent, with esophageal cancer representing the largest proportion. The distinct features of cluster 1 were primarily attributed to esophageal cancer. In contrast, cluster 2 was notably different from cluster 1, with a 5% prevalence of NSCLC and a relatively higher proportion of HNSCC and colorectal cancer. The AhR expression in macrophages within cluster 2 was noticeable higher than in cluster 1. Cluster 3, which had the highest NSCLC proportion, generally displayed a lower AhR expression than the other clusters. To precisely assess AhR expression in cancer and immune cells, we conducted quantitative statistical comparisons (**Figures 7F, G**). Although AhR was expressed in both the cytosol and nucleus of the tumor cells, its nuclear expression was generally higher. However, a similar expression pattern was observed in immune cells.

## 4 Discussion

We conducted analyses using hundreds of cores across five cancer types by employing mIHC, machine learning, and quantification methods. In contrast with conventional approaches, we utilized a high-throughput (HT) approach, yielding objective and quantitative results (**Supplementary Figure 1**). Additionally, using unbiased clustering, we analyzed the AhR expression patterns, identified the characteristics of three common clusters across cancer types, and examined their distribution in five solid tumors (**Figure 7**). One of the HT analysis advantages is its ability to quantitatively analyze AhR-expressing cells in the TME. Simultaneously, it allows the analysis of the cell nucleus and cytosol within and outside the tumor, enabling the statistical analysis of patterns. This yielded intriguing results summarizing the proteomic and cellular pathology-based outcomes that could not be achieved using methods such as single-cell RNA-seq. AhR expression is prominent in the TME, particularly in the nucleus. The AhR expression in tumors was notably higher than in immune cells in the TME. These findings are consistent with previous ones (14–17). The mIHC results for NSCLC showed that AhR was predominantly expressed in the nucleus in cancer cells, in accordance with our findings (18, 19). AhR is involved in detoxifying the environmental toxin polycyclic aromatic hydrocarbon, a tobacco smoke component. Thus, the smoking habits may influence AhR expression. No significant AhR expression differences between smokers and non-smokers have been found. Here, we did not investigate the potential impact of the smoking status on AhR expression because of the lack of information on the patient smoking status (11). Particularly, we observed that AhR was predominantly expressed in the nucleus in the five cancer types. AhR is expressed in the nucleus and performs various functions in the TME after interacting with ARNT. The increased presence of AhR is associated with drug resistance mechanisms due to an enhanced drug metabolism. Additionally, approximately 5,000 genes (20) regulated by AhR are highly relevant to epigenetic alterations (20, 21) and the differentiation associated with tumor heterogeneity (22–24). Although the analysis of 43 tumor cores done here may not be sufficient to draw definitive conclusions, the elevated cytosolic AhR expression found in NSCLC compared to that found in the other four cancer types is intriguing. AhR activates non-canonical signaling pathways in the cytosol (25). Furthermore, through signaling pathways involving protein kinase A (PKA), nuclear factor κB (NF-kB), and steroid receptor-coactivator-1 (Src1) in the cytosol, AhR might influence aspects such as cancer cell survival and resistance (26–29). Investigating how the AhR expression pattern in NSCLC differs from that in other cancer types is crucial.

AhR expression was not directly correlated with the tumor stage, grade, or metastasis, presenting a different pattern from those found previously (15, 30). Previously, a higher correlation with factors such as the stage and grade was reported in 25 patients with oral squamous cell carcinoma (30). These results indicate a different direction from that of our research findings. Such discrepancies may stem from the differences in patient tissues. For more reliable results, exploring the association between AhR activity and specific drug treatments, especially in patients with stage 3 or 4 cancer, would be valuable. In particular, patients with stage 3 or 4 cancer receiving anticancer drugs constitute an important population which was only studied to a low extent here, since we predominantly included stage 1 and 2 patients (90.06%). This result disparity is closely linked to the differences observed in previous studies.

Although AhR expression was predominantly high in cancer cells, it was only found in 2.12% and 0.44% of T cells and macrophages, respectively. Although AhR expression was also observed in Tregs, Treg infiltration varied depending on the tumor type, thereby influencing the results. AhR expression in immune cells is influenced by an increased kynurenine (Kyn) level induced by indoleamine 2,3-dioxygenase 1 (IDO1), which is the mechanism of action of anticancer agents targeting AhR. AhR expression in immune cells leads to a decreased immune activity due to Kyn, showing inhibitory effects on T-cell differentiation and anticancer functions, along with cytokine modulation (31). Additionally, AhR-high macrophages play a crucial role in increasing the levels of immunosuppressive cytokines, such as TGF-beta and IL-10, which are anti-inflammatory cytokines, in the TME (32). This implies a potential possibility for the polarization of M2 macrophages by these cytokines. Furthermore, AhR influences Treg differentiation and activation (33).

Although AhR expression differs among cancer types, AhR is generally expressed in most tumors. Interestingly, the expression pattern can be categorized into three clusters: Cluster 1, in which tumors exhibit a high AhR expression; cluster 3, characterized by relatively low AhR expression levels in tumors but a high expression in Tregs; and cluster 2, which represents a mixed pattern. This AhR expression pattern was observed across all five solid tumors analyzed here. The distinct AhR expression patterns observed across clusters suggest the potential application of AhR inhibitors in anticancer drug development and as predictive and prognostic biomarkers.

AhR expression has not been analyzed across various cancer types using mIHC. Although some studies have obtained pathological results from certain IHC analyses (34), large-scale results obtained using quantification methods are lacking. In particular, with the recent development of machine-learning techniques for analyzing pathological tissues, such analyses have become feasible. The nuclear or cytosolic AhR expression has been described (35–37). However, since these results were not obtained using multiplex analysis, ensuring the accuracy of the results obtained is challenging because the cells with a similar size and morphology to those of tumor cells may also be stained. Previous analyses of AhR expression in immune cells have often focused on specific immune cell types and have not been conducted using large clinical samples. Consequently, the results generated conceptual outcomes excluding the overall AhR expression distribution. In contrast, we comprehensively analyzed the heterogeneous AhR expression patterns in the TME. This approach allowed us to estimate the functional implications of AhR and identify the potential mechanisms of action for AhR-targeting drugs.

The limitations of this study are the inability to establish a correlation between AhR expression and immune checkpoint inhibitors, such as IDO and programmed death-ligand 1 (PD-L1). While we mainly focused on analyzing AhR expression and distribution, we failed to demonstrate their interaction with clinically significant mechanisms mediated by molecules such as IDO1, indoleamine 2,3-dioxygenase 2 (IDO2), tryptophan 2,3-dioxygenase (TDO), and PD-L1. Nevertheless, our data suggest a positive correlation between the increase in the amount of tumor-infiltrating lymphocytes (TILs) and TAMs and PD-L1 expression. Second, most tumors were obtained from surgical samples. Considering the tendency of AhR to react with chemical substances, a response to specific drugs is possible, especially since recent studies have reported an increase in AhR activity due to drugs such as chemotherapeutics or TKIs. Therefore, the frequency and increasing trend of AhR expression observed here may be more pronounced in patients treated with specific medications. Third, the limited clinical information available for the TMA prevents the direct validation of the patient outcomes. However, leveraging public databases, such as TCGA, could offer an opportunity to more comprehensively evaluate the impact of AhR on tumors. These issues should be addressed in future studies. Careful analysis is required to interpret AhR expression changes before and after anticancer treatment, especially if the transition from cluster 1 to clusters 2 or 3 is possible. Additionally, our observation of AhR expression in immune cells, particularly the significant increase observed in the stroma, led to hypotheses regarding the accessibility of various AhR ligands due to stromal vascular characteristics. Moreover, the high Kyn expression in macrophages may influence the stromal immune cell concentration. Experimental validation of these hypotheses is expected to contribute to a better understanding and interpretation of our findings.

AhR-targeting anticancer therapies are being actively developed. Particularly, small-molecule inhibitors such as BAY-241696 and IK-175 are currently being investigated in various solid tumors and have shown promising results (NCT05472506, NCT04200963, and NCT04999202). Recently developed AhR inhibitors, such as DA-4505, BAY-241696, and IK-175, have been presented at international scientific conferences during preclinical research. These drugs play a crucial role in immune cell activation in the TME. Notably, they are correlated with IDO, making biomarker and clinical-based mechanism of action analyses essential for novel drug development. The analysis of the AhR expression patterns conducted here is a significant milestone in this research field.

## 5 Conclusion

Our HT method employing mIHC and image cytometry based on AhR expression identified three distinct clusters. Cluster 1 exhibited high AhR expression in cancer cell nucleus, while cluster 3 showed the lowest. In cluster 2, stromal macrophage nucleus had the highest AhR expression. Cluster 1 was dominated by esophageal cancer, while cluster 2 had a higher proportion of HNSCC and colorectal cancer, with a small NSCLC presence. Cluster 3 had the highest NSCLC representation but lower AhR expression overall. By demonstrating different expression patterns of AhR in cancer cells and immune cells, our findings are anticipated to provide a fundamental basis for both biological and immunological research on drugs targeting AhR.

## Data availability statement

The original contributions presented in the study are included in the article/**Supplementary Material**. Further inquiries can be directed to the corresponding authors. The datasets generated for this study can be found in the Code Ocean: https://doi.org/10.24433/CO.1505908.v1.

## Ethics statement

All tissues were collected under the highest ethical standards with the donor being informed completely and with their consents. We followed standard medical care and protected the donors’ privacy. All human tissues were collected under HIPPA approved protocols. The studies were conducted in accordance with the local legislation and institutional requirements. The participants provided their written informed consent to participate in this study.

## Author contributions

DK: Conceptualization, Data curation, Formal analysis, Investigation, Methodology, Resources, Software, Validation, Visualization, Writing – original draft, Writing – review & editing. CL: Conceptualization, Data curation, Investigation, Methodology, Resources, Validation, Visualization, Writing – original draft, Writing – review & editing. YH: Conceptualization, Data curation, Investigation, Methodology, Resources, Validation, Visualization, Writing – original draft, Writing – review & editing. SP: Formal analysis, Resources, Writing – review & editing. HH: Writing – review & editing, Data curation, Resources, Software. KN: Resources, Validation, Writing – review & editing. MK: Methodology, Resources, Writing – review & editing. SY: Resources, Visualization, Writing – review & editing. SB: Investigation, Resources, Writing – review & editing, Validation. YK: Resources, Validation, Writing – review & editing. JH: Data curation, Resources, Writing – review & editing. SL: Investigation, Resources, Writing – review & editing. S-SK: Methodology, Resources, Writing – review & editing. MH: Resources, Validation, Writing – review & editing. SML: Resources, Validation, Writing – review & editing. JL: Resources, Validation, Writing – review & editing. JK: Formal analysis, Methodology, Project administration, Software, Writing – review & editing. BC: Funding acquisition, Resources, Writing – review & editing. K-HP: Conceptualization, Data curation, Formal analysis, Investigation, Methodology, Project administration, Resources, Software, Supervision, Validation, Visualization, Writing – original draft, Writing – review & editing.
