# Supplementary material for: Exploring aryl hydrocarbon receptor expression and distribution in the tumor microenvironment, with a focus on immune cells, in various solid cancer types
Source: Front Immunol. 2024 Apr 12;15:1330228. doi: 10.3389/fimmu.2024.1330228 (PMC11045933; doi:10.3389/fimmu.2024.1330228)
Supplement: Supplementary file 8 [file Image_1.pdf]

Fig. S1, Kim DK, Lee CY, Han YJ et al.

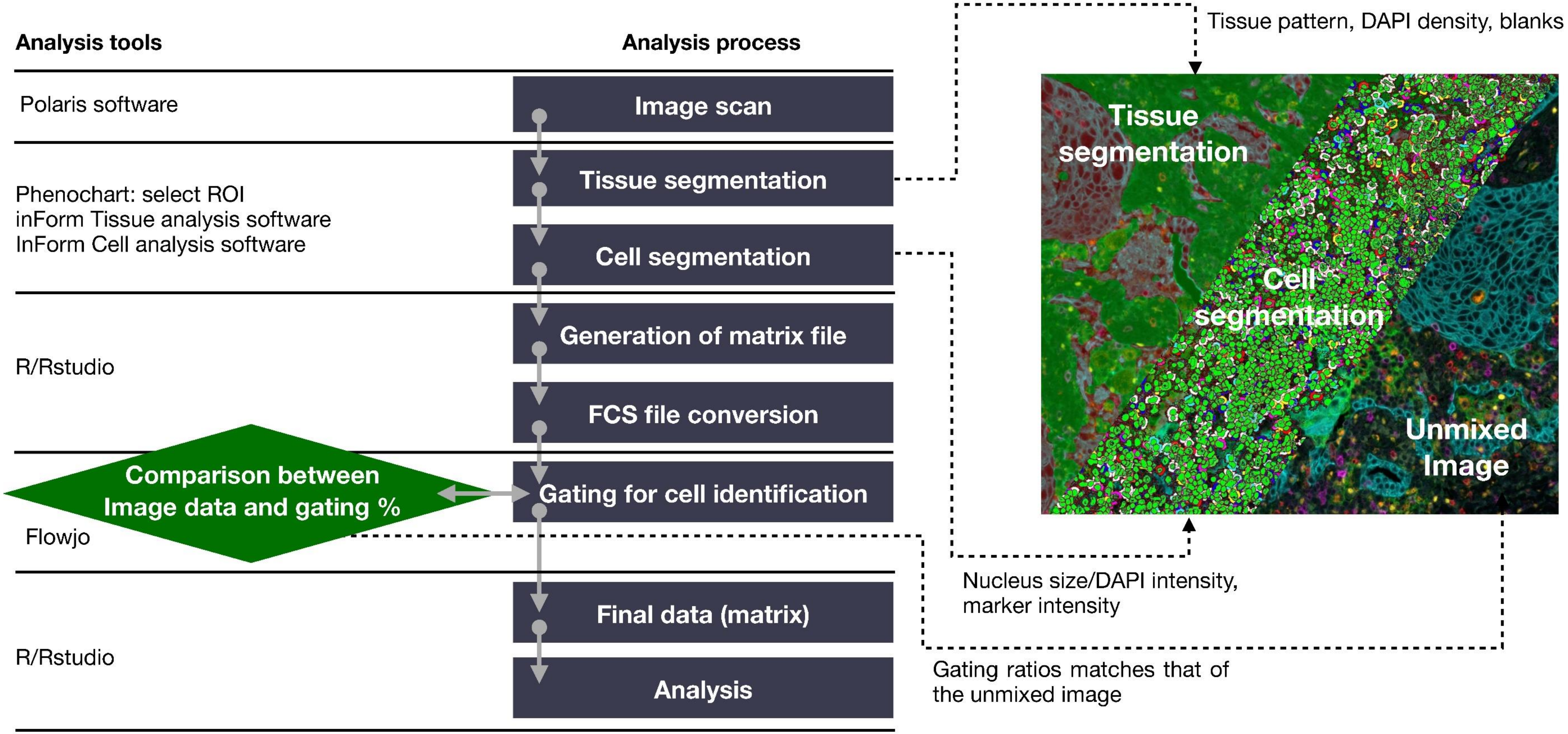

Figure S1. Schematic diagram of the softwares utilized throughout the investigation.

Fig. S2, Kim DK, Lee CY, Han YJ et al.

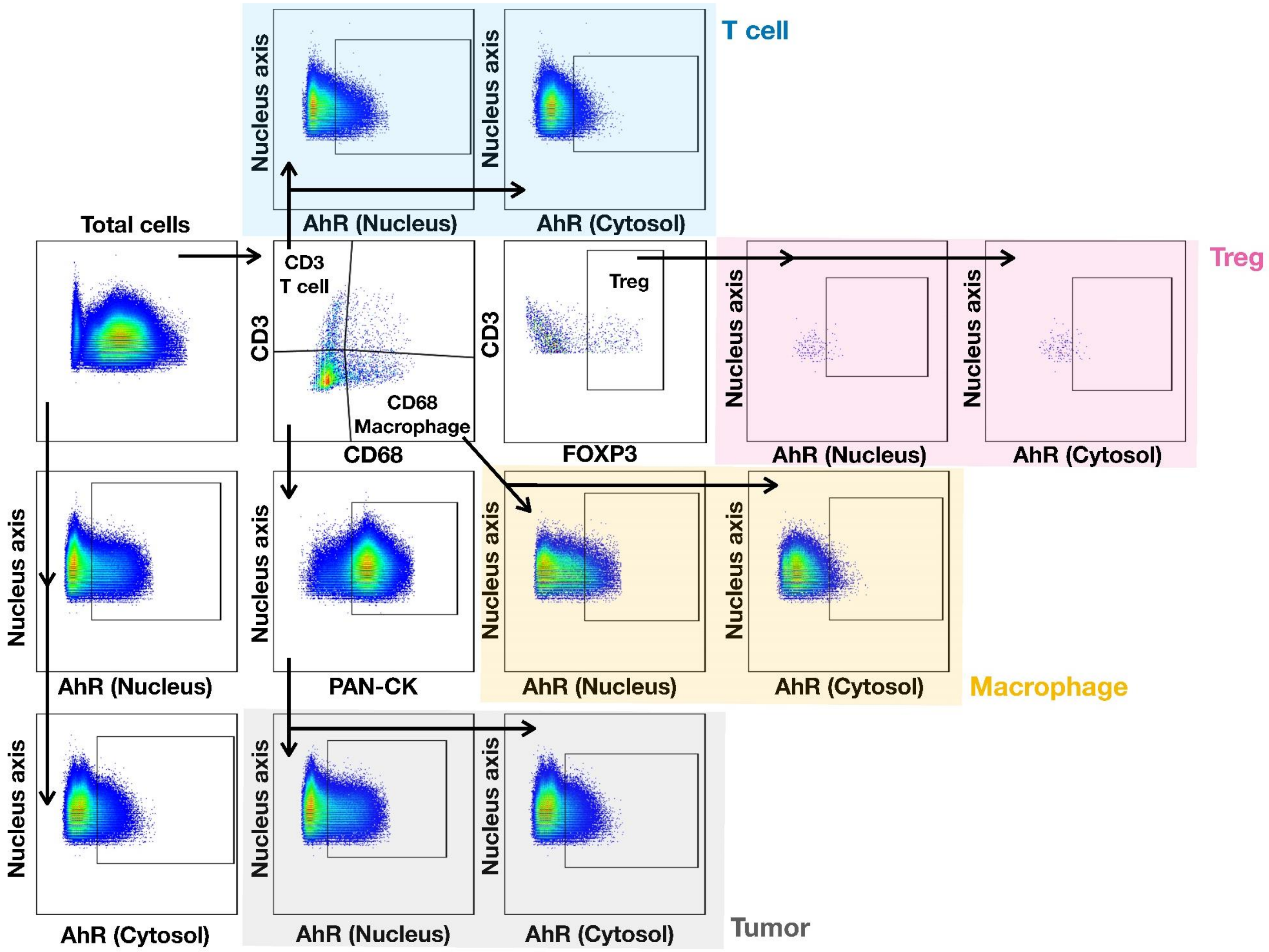

Figure S2. Gating strategy for cell phenotyping and AhR expression.

Fig. S3, Kim DK, Lee CY, Han YJ et al.

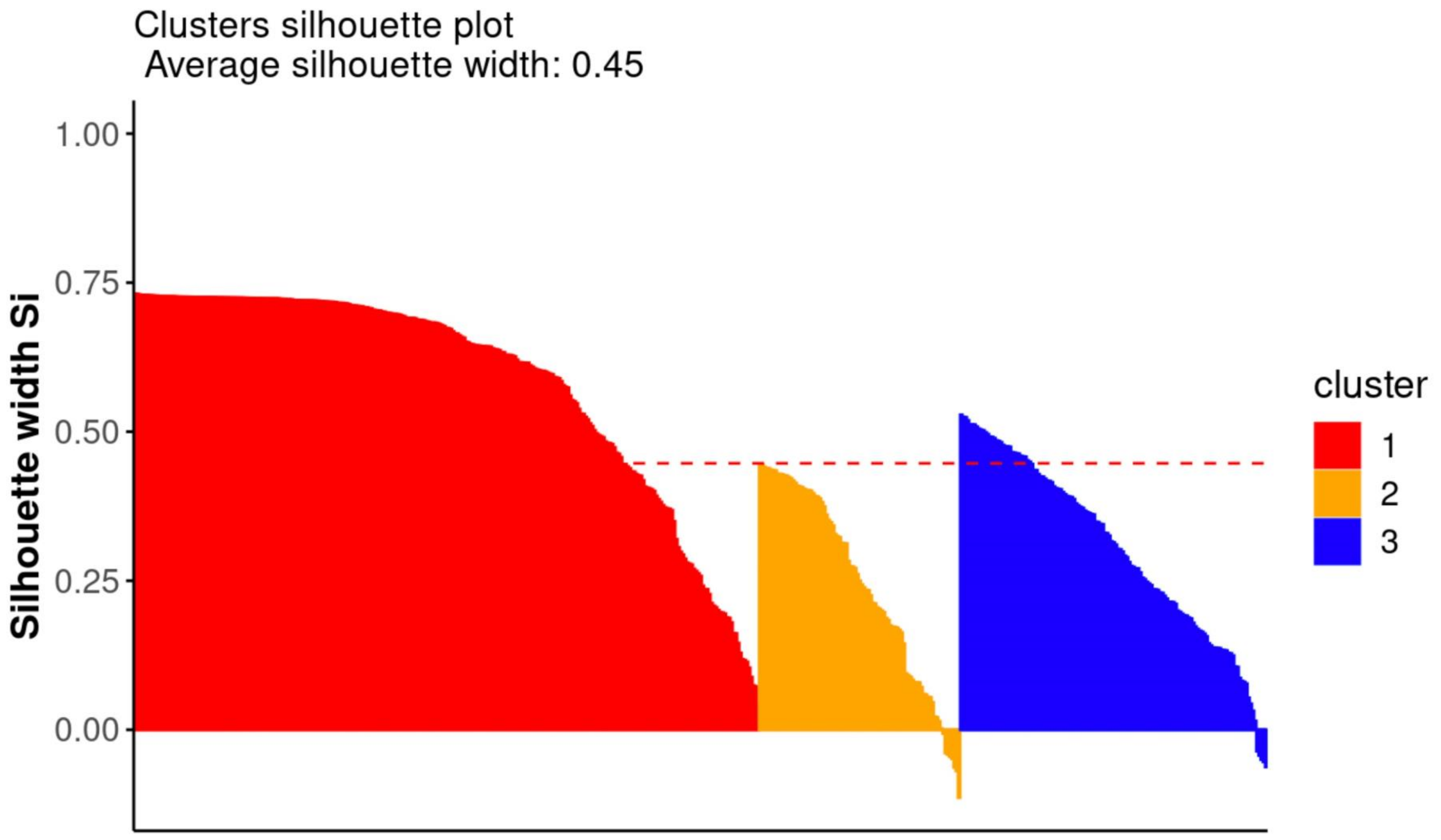

Figure S3. Silhouette plot illustrating a comprehensive comparison of five cancer types based on AhR expression using K-mean clustering.
